# Revisiting vSGLT: Non-Radioactive Characterization of the Vibrio Na^+^/Galactose Cotransporter by SURFE^2^R N1 Solid-Supported Membrane Electrophysiology

**DOI:** 10.3390/ijms27062790

**Published:** 2026-03-19

**Authors:** Natalia Ermolova, Thorsten Althoff, Ernest M. Wright, Jeff Abramson

**Affiliations:** Department of Physiology, David Geffen School of Medicine, University of California, Los Angeles (UCLA), Los Angeles, CA 90095, USA; nermolova@mednet.ucla.edu (N.E.); talthoff@mednet.ucla.edu (T.A.);

**Keywords:** *Vibrio parahaemolyticus* sodium–galactose transporter (vSGLT), electrogenic transport, solid-supported membrane electrophysiology, sodium–glucose coupling

## Abstract

The sodium–galactose cotransporter from *Vibrio parahaemolyticus* (vSGLT) was first cloned and functionally characterized by the laboratory of Ernest M. Wright in 2000, establishing a one-to-one Na^+^:sugar coupling stoichiometry and pioneering a bacterial model for human SGLTs. Here, we revisit vSGLT using solid-supported membrane electrophysiology on the Nanion SURFE^2^R N1, providing a modern, non-radioactive kinetic analysis of Na^+^-coupled sugar transport. Rapid transient currents were observed upon substrate application to proteoliposomes containing purified vSGLT. D-galactose elicited the largest Na^+^-dependent responses, followed by D-glucose and D-fucose, while no transport was observed in K^+^-based solutions. Apparent kinetic parameters recapitulate the overall trends observed in the original radiolabeled uptake assays, with *K*_m_(Na^+^) ≈ 18 mM and *K*_m_(gal) ≈ 9.8 mM. These findings validate the SURFE^2^R N1 SSM system as a quantitative, label-free method for Na^+^ symport characterization and demonstrate that vSGLT retains its canonical substrate selectivity and stoichiometry.

## 1. Introduction

Sodium-coupled cotransporters represent one of the most fundamental classes of secondary active membrane proteins [1]. Among these, sodium–glucose cotransporters (SGLTs) mediate the translocation of sugars across biological membranes using the electrochemical Na^+^ gradient [2,3]. The concept of Na^+^-coupled glucose transport was established through the pioneering research of Ernest M. Wright, whose work defined the molecular identity, transport stoichiometry, and kinetic principles of SGLTs [4].

A milestone of this effort was the cloning and characterization of the *Vibrio parahaemolyticus* sodium–galactose transporter (vSGLT), published in 2000 [5]. In proteoliposomes, vSGLT exhibited saturable, Na^+^-dependent uptake of D-galactose with a 1:1 Na^+^:sugar stoichiometry and a substrate preference resembling mammalian SGLTs. This bacterial homologue became central to structural and mechanistic studies, ultimately yielding the first crystal structures of an SGLT homologue [6,7], detailed biophysical characterization [8,9], and forming the basis for subsequent cryo-EM structures of human SGLT1 [10,11] and SGLT2 [12,13].

While the original characterization relied on radiolabeled sugar flux assays, advances in membrane electrophysiology now offer label-free alternatives. Solid-supported membrane electrophysiology (SURFE^2^R N1, Nanion) detects charge movements driven by transport activity with high temporal resolution, enabling direct interrogation of electrogenic coupling without radioactive isotopes [14,15]. Here, we utilize the SURFE^2^R N1 to re-examine vSGLT transport activity, comparing modern transient kinetic measurements with foundational uptake data.

## 2. Results

### 2.1. Substrate Specificity Recapitulates Classical vSGLT Selectivity

To characterize vSGLT transport activity, purified vSGLT was reconstituted into proteoliposomes and analyzed using solid-supported membrane electrophysiology (SURFE^2^R N1), which detects transient charge movements associated with substrate translocation. In each experiment, rapid buffer-exchange pulses containing defined sugars and cations were applied to proteoliposomes adsorbed onto the sensor surface, and the resulting transient peak currents (I_peak_) were analyzed after baseline correction.

**Figure 1:**
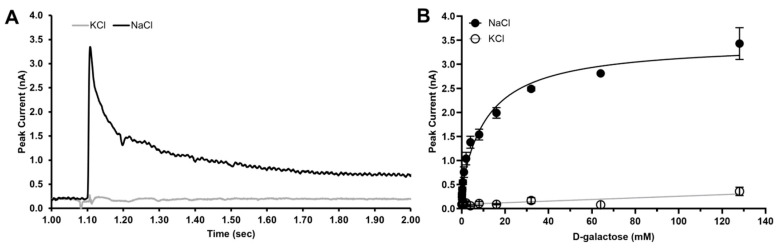
D-galactose transport measured by SURFE^2^R N1. (**A**) Representative transient current traces recorded on the same 3 mm sensor following application of 20 mM D-galactose in the presence of 150 mM NaCl (black) or 150 mM KCl (grey) at pH 7.5. (**B**) Peak transient currents measured as a function of D-galactose concentration in the presence of 150 mM NaCl (filled circles) or 150 mM KCl (open circles) at pH 7.5. Data represent mean ± SEM (NaCl *n* = 4, KCl *n* = 3). Curves were fit using three-parameter nonlinear regression.

D-galactose was chosen as the reference substrate to illustrate the analysis workflow. Initial recordings showed that D-galactose elicited the largest Na^+^-dependent transient currents among all sugars tested, confirming its role as the preferred substrate of vSGLT, in contrast to human SGLTs, which display higher affinity for D-glucose. Application of D-galactose in Na^+^-containing buffer induced rapid inward currents peaking at ~3–4 nA, followed by a biphasic decay consisting of a fast and a slow component. The fast component is attributed to charge movements associated with substrate binding, whereas the slower decay reflects charge movements coupled to transport turnover (Figure 1A).

Figure 1B presents the concentration-dependent responses measured in 150 mM NaCl (activating) and 150 mM KCl (non-activating) buffers at pH 7.5. Peak currents increased with D-galactose concentration in the presence of Na^+^ and saturated at higher substrate concentrations, whereas only minimal responses were observed in K^+^-based buffers. Peak currents were normalized to the maximal response and averaged across three independent sensors (*n* = 3), demonstrating that vSGLT-mediated transport activity measured by SSM electrophysiology is strictly Na^+^ dependent and recapitulates the canonical substrate selectivity originally defined by radiolabeled uptake assays.

**Figure 2:**
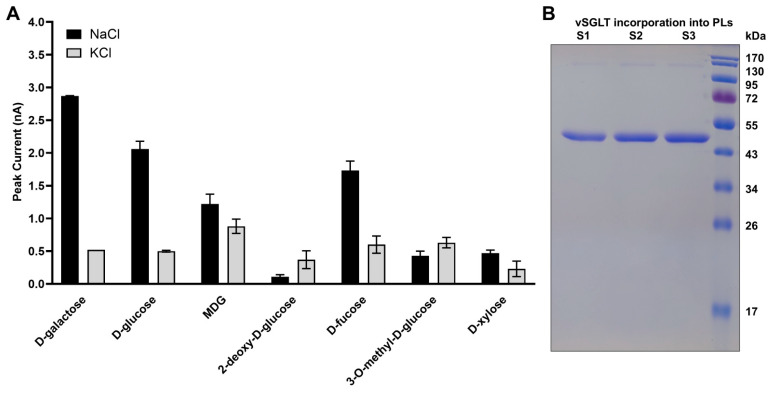
Substrate specificity of vSGLT measured by SURFE^2^R N1. (**A**) Peak transient currents recorded for different sugar substrates in the presence of 150 mM NaCl (dark gray) or 150 mM KCl (light gray). Experiments were performed at pH 7.5 using 3 mm sensors (*n* = 3) with a total sugar concentration of 128 mM. Bars represent mean ± SEM after normalization of each dataset to its maximal signal prior to averaging. (**B**) Coomassie Brilliant Blue-stained SDS–PAGE demonstrating incorporation of purified vSGLT into proteoliposomes for samples loaded onto three independent sensors (lanes S1–S3).

The same analysis was applied to all other sugars tested, including D-glucose, D-fucose, 2-deoxy-D-glucose, 3-O-methyl-D-glucose, and D-xylose. The resulting normalized Na^+^-dependent responses are summarized in Figure 2. Collectively, these data show that vSGLT preferentially transports D-galactose, followed by D-glucose and D-fucose, whereas D-xylose and 3-O-methyl-D-glucose elicit minimal responses, consistent with the substrate selectivity originally reported by Wright et al. [5].

### 2.2. vSGLT Exhibits Saturable Na^+^ Dependence

**Figure 3:**
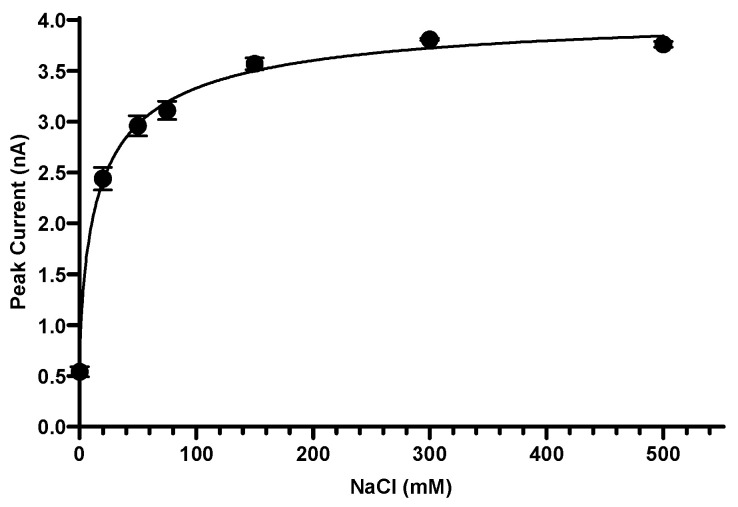
Na^+^-dependence of vSGLT transport activity. Peak transient currents were recorded as a function of NaCl concentration (0–500 mM) in the presence of 20 mM D-galactose at pH 7.5. Each dataset was recorded from a single 3 mm sensor, normalized to the maximal signal, and averaged across three independent sensors (*n* = 3). Currents measured in K^+^-containing buffers were subtracted from the corresponding Na^+^-dependent responses to isolate the Na^+^-specific component of transport. Data represent mean ± SEM. Curves were fit using four-parameter nonlinear regression.

Because vSGLT couples sugar transport to the Na^+^ gradient, we next examined how transport activity varies with sodium concentration. Using 20 mM D-galactose as the substrate, peak transient currents were recorded over a NaCl concentration range of 0–500 mM at pH 7.5. The resulting current–concentration relationship was saturable and approached a plateau at higher Na^+^ concentrations, consistent with Na^+^ binding driving transport activity (Figure 3). No measurable currents were detected under otherwise identical conditions in K^+^-containing buffers, confirming strict Na^+^ dependence of vSGLT transport.

Subtraction of K^+^ background signals from Na^+^-dependent responses yielded an apparent *K*_m_(Na^+^) ≈ 18.0 ± 3.6 mM, with a Hill coefficient of 0.7, consistent with a 1:1 Na^+^:sugar coupling stoichiometry. These results demonstrate that sodium binding is essential for transporter activation, consistent with a single high-affinity Na^+^ binding site driving transport (Figure 3).

These findings confirm that sodium binding is essential for transporter activation and that vSGLT retains strict Na^+^ specificity under the reconstituted assay conditions. The apparent Na^+^ affinity determined by SSM electrophysiology differs from that reported in radiolabeled uptake assays using [^14^C]D-galactose in proteoliposomes, which yielded a higher *K*_m_(Na^+^) of ~129 ± 8 mM with a Hill coefficient of 0.9. This difference likely reflects fundamental distinctions between the two assay modalities. Solid-supported membrane electrophysiology primarily reports on rapid electrogenic charge movements associated with early steps in the transport cycle, such as Na^+^ and substrate binding and associated conformational transitions, whereas radiolabeled uptake assays integrate net substrate accumulation over time and are sensitive to later rate-limiting steps, including substrate translocation and release. As a result, the Na^+^ dependence measured by SSM electrophysiology may reflect a higher-affinity binding or pre-steady-state step, while radiotracer uptake reports on the composite kinetics of the full transport cycle.

### 2.3. Sugar Concentration Dependence Confirms Na^+^-Coupled Transport

Having established the ionic requirement for transport, we next examined how substrate concentration influences vSGLT activity under saturating Na^+^ conditions (150 mM NaCl). Peak transient currents were recorded as a function of increasing D-galactose, D-glucose, or D-fucose concentration (0–128 mM) at pH 7.5, with parallel control measurements performed in 150 mM KCl.

**Figure 4:**
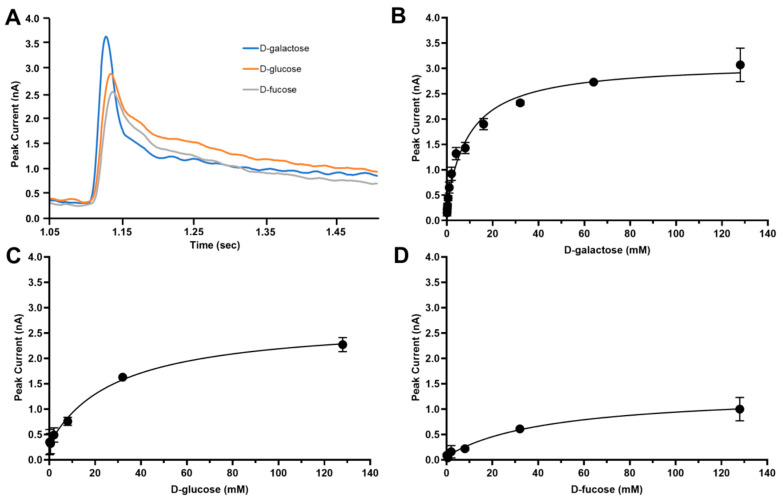
Sugar concentration dependence of vSGLT transport. (**A**) Representative transient current traces recorded on the same 3 mm sensor for D-galactose (blue), D-glucose (orange), and D-fucose (gray) in the presence of 150 mM NaCl at pH 7.5. (**B**) D-galactose, (**C**) D-glucose, and (**D**) D-fucose concentration-dependent Na^+^-specific peak currents measured at pH 7.5. Each dataset was recorded from a 3 mm sensor, normalized to the maximal signal, and averaged across three independent sensors (fucose NaCl *n* = 4, all others *n* = 3). Na^+^-specific currents were obtained by subtracting responses measured in K^+^-containing buffers from the corresponding Na^+^-dependent responses. Data represent mean ± SEM. Curves were fit using three-parameter nonlinear regression.

For each substrate, current amplitudes increased with sugar concentration and approached saturation at higher concentrations, yielding hyperbolic concentration–response relationships characteristic of saturable transport kinetics (Figure 4). Subtraction of currents measured in K^+^-containing buffers from those recorded in Na^+^-containing buffers isolated the Na^+^-specific transport component. Fits to these data yielded apparent Michaelis–Menten constants of approximately 9.8 mM for D-galactose, 29.2 mM for D-glucose, and 44.9 mM for D-fucose, consistent with the substrate preference observed in earlier measurements [5]. Together, these results indicate that the recorded transient currents reflect electrogenic Na^+^/sugar symport and that vSGLT exhibits distinct apparent substrate affinities depending on the sugar moiety.

## 3. Discussion

This work revisits vSGLT—the first SGLT homologue cloned—using modern, label-free electrophysiology. Using solid-supported membrane electrophysiology, we reproduce the key functional features originally established by Ernest M. Wright and colleagues, including saturable Na^+^ dependence, a strong preference for D-galactose, and electrogenic 1:1 coupling of Na^+^ and sugar transport. Together, these results reinforce the canonical mechanistic framework for vSGLT function that emerged from early radiolabeled uptake studies.

While the apparent Na^+^ affinity measured by SSM electrophysiology differs from that reported in radiotracer-based transport assays, both approaches support a single Na^+^ binding event tightly coupled to sugar transport. These differences likely reflect the distinct kinetic steps probed by the two methodologies, with SSM electrophysiology reporting on rapid electrogenic events associated with binding and early conformational transitions, and radiotracer uptake integrating net transport over the full catalytic cycle. In this context, the two techniques should be viewed as complementary rather than directly equivalent.

Beyond revisiting vSGLT function, the present work highlights the continued utility of vSGLT as a benchmark system for studying Na^+^-coupled transport using contemporary biophysical tools. By extending classical measurements into the realm of real-time, high-resolution electrophysiology, this study provides a direct link between foundational transport models and modern experimental approaches. In doing so, it also serves as a tribute to the lasting scientific legacy of Ernest Wright, whose integration of biophysics, physiology, and structure continues to shape the field of membrane transport.

## 4. Materials and Methods

### 4.1. Expression and Purification of vSGLT

vSGLT was expressed in *E. coli*, purified via affinity- and size-exclusion chromatography, and concentrated in buffer containing decyl-maltoside detergent prior to reconstitution [5]. Briefly, XL1-Blue transformed with vSGLT_A423C in pJExpress plasmid (DNA2.0, Newark, CA, USA) were grown in Terrific Broth (Teknova, Hollister, CA, USA) supplemented with kanamycin at 37 °C, 220 rpm to an OD = 1.8. Protein expression was induced by the addition of 0.75 mM IPTG and the temperature was lowered to 33 °C. After 2.5 h cells were pelleted and resuspended in lysis buffer (50 mM Tris pH 8.0, 100 mM NaCl, 5 mM 2-mercaptoethanol (BME), 0.1 mM PMSF) at a ratio of 3 mL/g cells. Cell lysis was accomplished by three passes through an EmulsiFlex-C3 cell extruder (Avestin Inc., Ottawa, ON, Canada). Debris was removed by centrifugation for 35 min at 15,000× *g* and membranes were pelleted from the supernatant by centrifugation at 302,000× *g* for 2 h. Membranes were resuspended in resuspension buffer at a ratio of 10 mL/g membranes (70 mM Tris pH 8.0, 150 mM NaCl, 20 mM Imidazole, 4 mM Na_3_Citrate, 6% Glycerol, 5 mM BME, 0.1 mM PMSF) and solubilized with 2% n-decyl-β-maltoside (DM) (GLYCON Biochemicals GmbH, Luckenwalde, Germany) by stirring for 3 h at 4 °C. The sample was clarified by centrifugation at 53,300× *g* for 1 h and subsequently passed through a Ni-NTA HisTrap FF affinity column (Cytiva, Malborough, MA, USA), equilibrated in resuspension buffer with 0.174% DM. The column was washed with 10 column volumes (CV) of resuspension buffer with 0.174% DM, followed by 10 CV of resuspension buffer with 35 mM imidazole, 0.174% DM. vSGLT was eluted with a gradient to 200 mM imidazole, concentrated in a 50 kDa Amicon Ultra-4 (Millipore, Burlington, MA, USA) and injected onto a Superdex 200 10/300 GL size exclusion column (Cytiva, Malborough, MA, USA) equilibrated with SEC buffer (25 mM Tris pH7.4, 150 mM NaCl, 0.02 mM EDTA, 2 mM BME, and 0.174% DM). Peak fractions containing vSGLT were pooled and concentrated to ~15 mg/mL.

### 4.2. Reconstitution into Proteoliposomes

Purified vSGLT was reconstituted into liposomes composed of *E. coli* Polar Lipid (Avanti Polar Lipids Inc., Alabaster, AL, USA) at a lipid-to-protein ratio of 10:1. Lipids were dissolved in chloroform and subsequently dried into a thin film under a stream of nitrogen gas. Dried lipids were resuspended in water at a concentration of 20 mg/mL and sonicated for four minutes. For reconstitution, 3.6 mg of lipids were first mixed with DM (final concentration 1%) and KPi, pH 7.5 (final concentration 150 mM). Finally, 360 µg of the protein was added. The reaction mix was incubated for 30 min on ice. Then, 100 µL were injected into rapidly stirring 60 mL of 150 mM KPi, pH 7.5. Liposomes were collected by centrifugation at 200,000× *g* for 3 h at 4 °C. The supernatant was completely removed and the pelleted proteoliposomes were resuspended in 180 µL of 150 mM KPi buffer. Before use, proteoliposomes were subjected to six freeze–thaw cycles in liquid nitrogen and a water bath at 32 °C. Protein incorporation was confirmed by SDS-PAGE.

### 4.3. SURFE^2^R N1 Sensor Preparation

Sensors were prepared according to published protocols [16]. 3 mm SURFE^2^R sensors were first thiolized with 0.5 mM 1-octadecanethiol (in 2-propanol) overnight. The next day, the sensors were washed three times each with 2-propanol and water. After drying, 2.0 µL of 7.5 mg/mL 1,2-diphytanoyl-sn-glycero-3-phosphocholine (DPhP) in n-decane and 50 µL of Non-Activating Buffer (25 mM Tris, pH7.5; 128 mM mannitol) were applied and incubated at room temperature for 3 h. The sensors were then rinsed with Non-activating Buffer before loading with 50 µL Non-Activating (NA) Buffer (50 mM Tris pH7.5; 1 M KCl; 20 mM mannitol). Before use, proteoliposomes were subjected to six freeze/thaw cycles, diluted 1:10 with 150 mM KPi, pH7.5 and sonicated 3 times for 10 s in a water bath. 10 µL of diluted proteoliposomes were added onto the sensor, followed by centrifugation at 3000× *g* for 30 min.

### 4.4. SURFE^2^R N1 Electrophysiology

Solid-Supported Membrane (SSM) electrophysiology measurements were carried out on a SURFE^2^R N1 (Nanion Technologies GmbH, Munich, Germany) at room temperature [17]. Sensor chips were loaded into the measuring chamber of the SURFE^2^R N1 and subjected to automated sequences of buffer changes. Each series of buffer exchanges was repeated three times per sensor. Non-activating (NA) Buffer was applied for 1 s, followed by 1 s of activating (A) Buffer. The reaction was stopped by 1 s application of Non-activating Buffer and an additional 1s rinse with Non-activating Buffer. Each buffer exchange sequence was repeated three times. To determine the affinity for different sugars, the Activating Buffer contained 25 mM Tris, pH7.5; 150 mM NaCl and 0–128 mM sugar (D-galactose, D-glucose or D-fucose). D-mannitol was used to adjust the osmolarity in sugar-containing Activating Buffers and replaced the sugar in Non-activating Buffer. To measure non-specific transport, experiments were conducted with buffers containing 150 mM KCl instead of NaCl. Sugar selectivity was determined using a constant sugar concentration of 128 mM sugar for Activating Buffer or 128 mM mannitol for Non-activating Buffer with either 150 mM NaCl or 150 mM KCl. To determine the affinity for Na^+^, Activating Buffers contained 25 mM Tris, pH7.5; 20 mM galactose and 0–500 mM NaCl with KCl added to maintain osmolarity. In Non-activating Buffer, galactose was replaced with mannitol. In the control experiments NaCl was replaced with KCl in the buffers.

### 4.5. Data Analysis

Currents were recorded in real time using the SURFE2R N1 Control Software v. 1.6.0.1. Peak currents were extracted and subsequently plotted in Graphpad Prism 10 (GraphPad Software, Boston, MA, USA). Background signals in K^+^ were subtracted from Na^+^ signals. Nonlinear regression was used to estimate *K*_m_ and Hill coefficients. *n* = 3 sensors per condition with three repetitions of the buffer exchange series per sensor. Regression models were selected based on the experimental design for each dataset.

## 5. Conclusions

SURFE^2^R N1 solid-supported membrane electrophysiology provides a robust, radioisotope-free platform for high-resolution functional analysis of vSGLT, faithfully recapitulating the core kinetic features, ion dependence, and substrate selectivity originally defined by Ernest Wright and colleagues. By directly measuring electrogenic Na^+^/sugar symport in a reconstituted system, this approach complements classical radiotracer assays and offers new insight into early transport-cycle events. Together, these results establish SSM electrophysiology as a powerful and broadly applicable method for the functional characterization of sodium-coupled transporters.

## Data Availability

The original contributions presented in this study are included in the article. Further inquiries can be directed to the corresponding author.

## References

[1] Abramson J., Wright E.M. (2009). Structure and function of Na(+)-symporters with inverted repeats. Curr. Opin. Struct. Biol..

[2] Wright E.M., Turk E. (2004). The sodium/glucose cotransport family SLC5. Pflug. Arch. Eur. J. Physiol..

[3] Wright E.M., Loo D.D., Hirayama B.A. (2011). Biology of human sodium glucose transporters. Physiol. Rev..

[4] Wright E.M., Loo D.D.F. (2021). Active Glucose Transport 2020 and Beyond. Function.

[5] Turk E., Kim O., le Coutre J., Whitelegge J.P., Eskandari S., Lam J.T., Kreman M., Zampighi G., Faull K.F., Wright E.M. (2000). Molecular characterization of *Vibrio parahaemolyticus* vSGLT: A model for sodium-coupled sugar cotransporters. J. Biol. Chem..

[6] Faham S., Watanabe A., Besserer G.M., Cascio D., Specht A., Hirayama B.A., Wright E.M., Abramson J. (2008). The crystal structure of a sodium galactose transporter reveals mechanistic insights into Na+/sugar symport. Science.

[7] Watanabe A., Choe S., Chaptal V., Rosenberg J.M., Wright E.M., Grabe M., Abramson J. (2010). The mechanism of sodium and substrate release from the binding pocket of vSGLT. Nature.

[8] Paz A., Claxton D.P., Kumar J.P., Kazmier K., Bisignano P., Sharma S., Nolte S.A., Liwag T.M., Nayak V., Wright E.M. (2018). Conformational transitions of the sodium-dependent sugar transporter, vSGLT. Proc. Natl. Acad. Sci. USA.

[9] Khan F., Elgeti M., Grandfield S., Paz A., Naughton F.B., Marcoline F.V., Althoff T., Ermolova N., Wright E.M., Hubbell W.L. (2023). Membrane potential accelerates sugar uptake by stabilizing the outward facing conformation of the Na/glucose symporter vSGLT. Nat. Commun..

[10] Niu Y., Cui W., Liu R., Wang S., Ke H., Lei X., Chen L. (2022). Structural mechanism of SGLT1 inhibitors. Nat. Commun..

[11] Han L., Qu Q., Aydin D., Panova O., Robertson M.J., Xu Y., Dror R.O., Skiniotis G., Feng L. (2022). Structure and mechanism of the SGLT family of glucose transporters. Nature.

[12] Hiraizumi M., Akashi T., Murasaki K., Kishida H., Kumanomidou T., Torimoto N., Nureki O., Miyaguchi I. (2024). Transport and inhibition mechanism of the human SGLT2-MAP17 glucose transporter. Nat. Struct. Mol. Biol..

[13] Niu Y., Liu R., Guan C., Zhang Y., Chen Z., Hoerer S., Nar H., Chen L. (2022). Structural basis of inhibition of the human SGLT2-MAP17 glucose transporter. Nature.

[14] Pintschovius J., Fendler K., Bamberg E. (1999). Charge translocation by the Na+/K+-ATPase investigated on solid supported membranes: Cytoplasmic cation binding and release. Biophys. J..

[15] Bazzone A., Körner A., Meincke M., Bhatt M., Dondapati S., Barthmes M., Kubick S., Fertig N. (2022). SSM-based electrophysiology, a label-free real-time method reveals sugar binding & transport events in SGLT1. Biosens. Bioelectron..

[16] Bazzone A., Barthmes M. (2020). Functional Characterization of SLC Transporters Using Solid Supported Membranes. Methods Mol. Biol..

[17] Bazzone A., Zerlotti R., Barthmes M., Fertig N. (2023). Functional characterization of SGLT1 using SSM-based electrophysiology: Kinetics of sugar binding and translocation. Front. Physiol..

